# Predicting the chemical space of fungal polyketides by phylogeny-based bioinformatics analysis of polyketide synthase-nonribosomal peptide synthetase and its modification enzymes

**DOI:** 10.1038/s41598-020-70177-w

**Published:** 2020-08-11

**Authors:** Atsushi Minami, Takahiro Ugai, Taro Ozaki, Hideaki Oikawa

**Affiliations:** Department of Chemistry, Faculty of Science, Hokkaido University, Sapporo, 060-0810 Japan

**Keywords:** Biochemistry, Computational biology and bioinformatics

## Abstract

Fungal polyketide synthase (PKS)–nonribosomal peptide synthetase (NRPS) hybrids are key enzymes for synthesizing structurally diverse hybrid natural products (NPs) with characteristic biological activities. Predicting their chemical space is of particular importance in the field of natural product chemistry. However, the unexplored programming rule of the PKS module has prevented prediction of its chemical structure based on amino acid sequences. Here, we conducted a phylogenetic analysis of 884 PKS–NRPS hybrids and a modification enzyme analysis of the corresponding biosynthetic gene cluster, revealing a hidden relationship between its genealogy and core structures. This unexpected result allowed us to predict 18 biosynthetic gene cluster (BGC) groups producing known carbon skeletons (number of BGCs; 489) and 11 uncharacterized BGC groups (171). The limited number of carbon skeletons suggests that fungi tend to select PK skeletons for survival during their evolution. The possible involvement of a horizontal gene transfer event leading to the diverse distribution of PKS–NRPS genes among fungal species is also proposed. This study provides insight into the chemical space of fungal PKs and the distribution of their biosynthetic gene clusters.

## Introduction

Fungi are a prolific source of polyketides (PKs), which display remarkable structural diversity and biological activity. Representative examples include the cholesterol-lowering drug lovastatin, antifungal drug griseofulvin, immunosuppressant drug mycophenolic acid, and phytopathogenic virulence factor T-toxin. Their common polyketide backbone is constructed by two types of polyketide synthases (PKSs): non-reducing PKS (NR–PKS) and highly reducing PKS (HR–PKS)^1–3^. Among them, HR–PKS has a single PKS module composed of ketosynthase (KS), malonyl-CoA:ACP transacylase (AT), dehydratase (DH), C-methyltransferase (C-MeT), active/inactive enoylreductase (ER/ER^0^), ketoreductase (KR), and acyl carrier protein (ACP) domains. The function of the inactive ER^0^ domain is complemented by a trans-acting ER (t-ER) auxiliary protein^1,4^. The PKS module acts iteratively to synthesize a structurally diverse polyketide backbone according to its inherent programming rule. HR-PKS includes PKS–nonribosomal peptide synthetase (NRPS) hybrids, which are fusion proteins of the PKS module and NRPS module comprising the condensation (C), adenylation (A), thiolation (T), and off-loading (DKC or R) domains as a built-in release mechanism for the polyketide chain^1,2^.

The wealth of fungal genome sequences deposited in public databases has enabled identification of biosynthetic gene clusters (BGCs) containing PKS genes using bioinformatics tools such as SMURF^5^ and AntiSMASH^6^. Phylogenetic analysis of these PKSs, especially when focusing on the KS domain instead of the PKS itself, has been used to predict sequential diversity^7–9^, PKS gene type^7–9^, and evolutionary processes^10^. However, to our knowledge, bioinformatics studies specifically investigating the relationship between the genealogy and the chemical structure of PKs are scarce^11^.

**Scheme 1:**
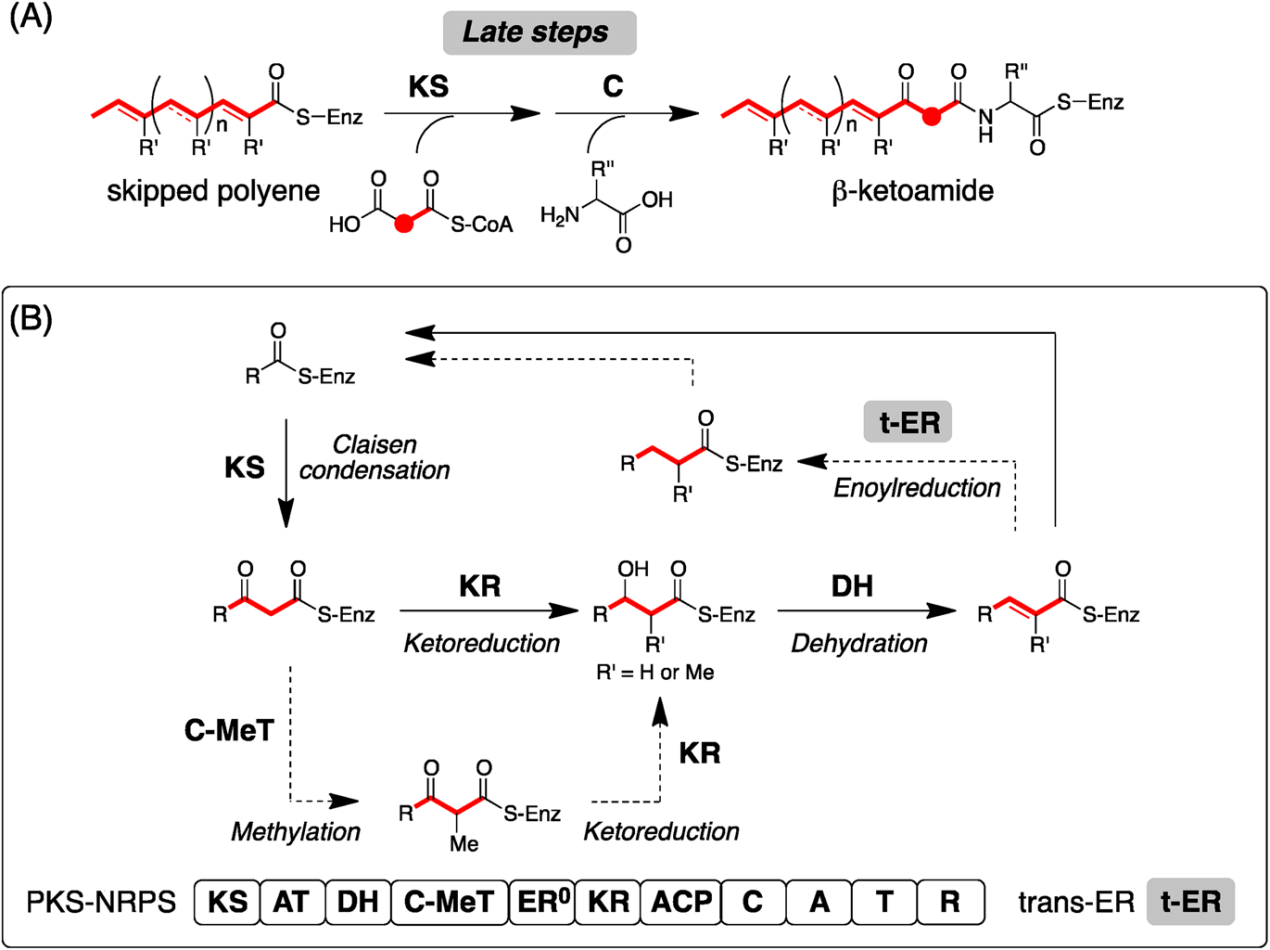
(**A**) Late biosynthetic steps to synthesize a β-ketoamide. (**B**) Chain elongation mechanism to synthesize a skipped polyene intermediate. Dotted lines show the optional pathways in the biosynthesis of most hybrid NPs.

Fungal PKS–NRPS hybrids (hybrids) are relatively a small family of HR–PKSs. This is consistent with the fact that a single fungal strain has a limited number of hybrid genes (less than three) when compared with the HR-PKS genes in its genome^12^. These hybrids participate in the biosynthesis of hybrid natural products (NPs), such as 2-pyridones^13^, cytochalasans^14,15^, and tetramate decalins^16^. Accumulation of structural information on hybrid NPs, in addition to putative biosynthetic gene clusters of hybrid NPs, provides opportunities to predict NP structures produced by a target BGC. The functional analysis of BGCs for structurally different hybrid NPs has accelerated their structure prediction. Recent biosynthetic studies have revealed that the PKS module basically gives a β-ketoamide intermediate via a skipped polyene with no oxygen functionality. During the chain elongation processes (Scheme 1), the KS domain essentially acts to synthesize a β-keto intermediate, which is subjected to a subsequent C-methylation by C-MeT to afford a methyl-substituted intermediate. This C-methylation process is optional, thereby affording structural variation of the resultant polyketide chain. Methylated/non-methylated β-keto intermediates undergo ketoreduction followed by dehydration to afford an α,β-unsaturated intermediate, which is reduced by t-ER. As in the case of C-methylation, the enoylreduction is also an optional process in the biosynthesis of most hybrid NPs. The number of chain elongation processes is inherently programmed by the PKS module. Consequently, structural differences resulting from the resultant polyketide (PK) chain have been identified in the chain length and the methylation patterns as well as the position of a double bond (Figure S1). The PKS-tethered intermediate is subjected to condensation with a malonyl-CoA followed by an amino acid moiety to give a β-ketoamide intermediate (Scheme 1). Two homologous hybrids, TenS and DmbS, were reported to produce structurally related β-ketoamide intermediates, indicating that the polyketide chain synthesis is inherently programmed in the amino acid sequence of the hybrid^17^. Based on these results, we hypothesized that homologous hybrids produce structurally related PK backbones. To examine our hypothesis, in this study, we conducted a phylogenetic analysis of fungal PKS-NRPS hybrids deposited in a public database and classified them to understand the structures of their products.

## Results

### Phylogeny-based classification of hybrids

We screened PKS genes from a public database (1,462 fungi) using the KS [Pfam protein family: ketoacyl-synt, PF00109] and AT [Acyl_transf_1, PF00698] domains to construct a PKS gene dataset including NR-PKS genes (total number: 3,311) and HR-PKS genes (6,709). We then eliminated the NR- and HR-PKS genes by focusing on a condensation domain (Condensation, PF00668) and re-constructed the dataset to comprise 1,419 hybrids. To ensure the inclusion of meaningful samples for phylogenetic analysis, we excluded highly homologous hybrids found in the same species, which are strongly expected to synthesize the same PK intermediate, and included the recently characterized hybrids. The final dataset included 884 sequences derived from Dothideomycetes, Eurotiomycetes, Lecarnoromycetes, Leotiomycetes, Orbiliomycetes, Pezizomycetes, and Sordariomycetes. No hybrid genes were identified in the other two subphyla of Ascomycota, Taphrinomycotina, and Saccharomycotina, as reported in previous bioinformatics analysis^8^. On the contrary, only 5 hybrids could be found in Basidiomycota fungi. This distribution bias suggested that, among Ascomycota fungi, Pezizomycotina is a major producer of biologically active PKs.

**Figure 1:**
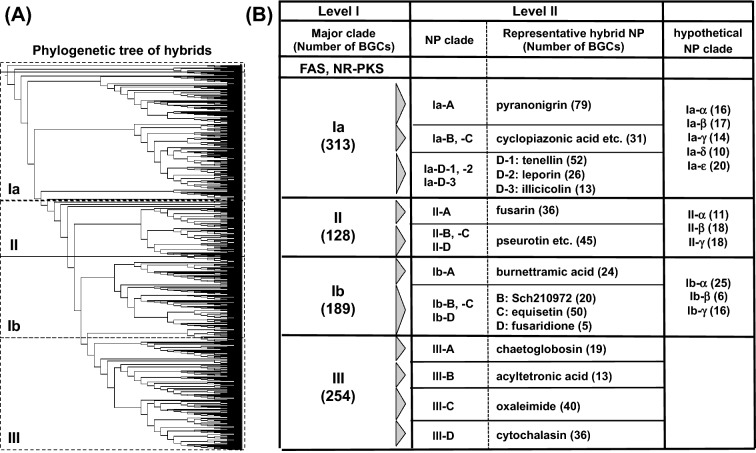
(**A**) Phylogenetic tree of fungal PKS-NRPS hybrids; (**B**) summary of BGC classification by searching for the key modification enzymes. The four major clades, Ia, Ib, II, and III, include NP clades. The number of BGCs classified into each NP clade is denoted in parentheses.

**Figure 2:**
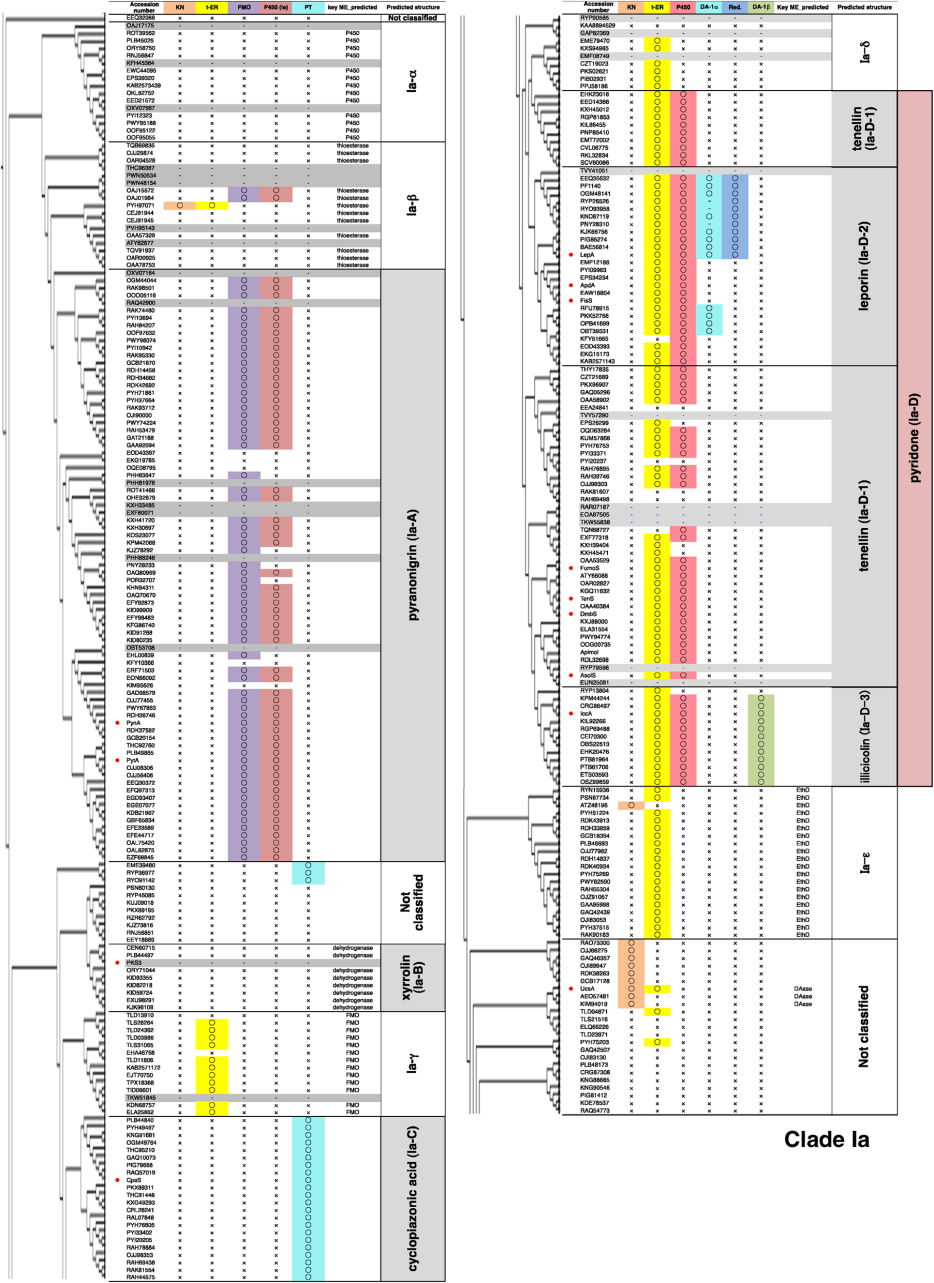
Enlarged view of the phylogenetic tree around hybrids for synthesizing 2-pyridone containing hybrid NPs (Clade Ia). Functionally characterized hybrids are marked by red circles. The modification enzyme genes located adjacent to each hybrid gene are described in the right side of the phylogenetic tree. Hybrid genes lacking enough sequence data are highlighted in grey colour.

For the phylogenetic analysis of the fungal PKS-NRPS hybrids (whole sequence of PKS-NRPS), we integrated the fungal NR-PKS (19 sequences) and fungal type I FAS (6 sequences) into the dataset (Table S1). The FAS, NR-PKS, and the hybrids were clustered into different groups (Fig. 1A). According to our hypothesis, we initially checked whether functionally characterized hybrids that synthesize structurally related PKs fall into the same clade in the hybrid phylogeny. We focused on two hybrids, EqxS and ChggA, which produce the same PK backbone with a C16 chain and a terminal triene moiety (Figure S1), and found that they form different groups. Similarly, FsdS and Fus1, which produce structurally related PKs (Figure S1), also form different groups. These results suggested that there is no relationship between the function of hybrids and their phylogenetic classifications. On the other hand, when focusing on the NP structure biosynthesized from functionally characterized hybrids, we found that phylogenetically related hybrids synthesize structurally and biosynthetically related NPs. For example, functionally characterized hybrids that synthesize 2-pyridone containing hybrid NPs such as aspyridone (hybrid name: ApdA^18^), tenellin (TenS^19^), desmethylbassianin (DmbS^20^), didymellamide (AsolS^21^), leporin (LepA^22^), and illicicolin (IccA^23^), are located in a specific region defined as the pyridone family clade (Ia-D) (Fig. 2). Considering that the common 2-pyridone structure is constructed by the action of modification enzymes, we focused on searching modification enzyme genes locating adjacent to the hybrid gene.

### Hybrid gene cluster analysis

#### Identification of the pyridone clade

**Scheme 2:**
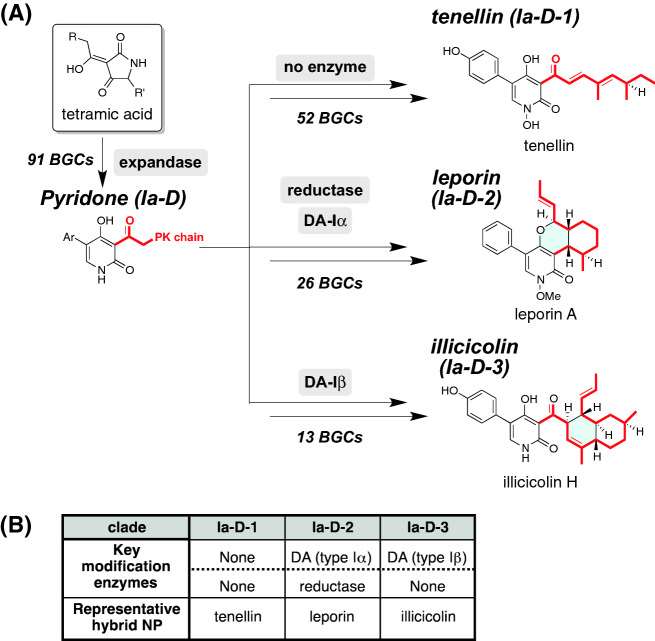
(**A**) Proposed biosynthetic pathways of hybrid NPs produced by the pyridone clade_BGCs. The common biosynthetic intermediate is the 2-pyridone derivative biosynthesized by the action of expandase; (**B**) summary of key modification enzymes to categorize three NP clades; tenellin, leporin, and illicicolin. Detailed biosynthetic schemes are summarized in Supplementary Information.

Hybrid NPs containing 2-pyridone are commonly biosynthesized from a tetramic acid intermediate (Scheme 2). Their oxidative ring expansion is catalysed by a cytochrome P450^24^, which is named as an expandase in this paper. As this modification is unique in the biosynthesis of this family member of hybrid NPs, we searched the expandase gene focusing on the 20 kbp flanking region of the hybrid gene, which is regarded as a BGC, and found that 84 hybrids accompany an expandase gene. Among them, 76 hybrids including seven known hybrids such as TenS form a distinct pyridone clade (final classification: Ia-D) composed of 91 hybrids, indicating that the corresponding BGCs might participate in the biosynthesis of 2-pyridone derivatives. For a simple comprehension of the relationship between the clade and hybrid NP, we used the term “NP clade” to denote the hybrid NP, which includes BGC groups possessing a similar set of key modification enzymes (Tables S2, S3, S4, S5).

The 2-pyridone derivatives constructed by the action of the expandase undergo subsequent modifications leading to leporin and illicicolin, which have polycyclic structures derived from the polyketide chain. Previous biosynthetic studies have revealed the key modification enzymes for synthesizing these polycyclic structures^23,25^ (Schemes 2B and S1). An additional modification enzyme (ME) search categorized the BGCs of the pyridone clade into three NP clades; tenellin clade (Ia-D-1, 52), leporin clade (Ia-D-2, 26), and illicicolin clade (Ia-D-3, 13) (Schemes 2 and S1, Fig. 1, and Table S2). Detailed features of Diels-Alderases used for the modification enzyme search are discussed in the following section. Notably, as in the case of didymellamide^21^, a spontaneous [4 + 2] cycloaddition also affords the same decalin skeleton. Therefore, lack of the DAase gene in the target BGC does not mean that the corresponding hybrid NPs have a linear PK chain.

#### Identification of NP clades for pyranonigrin, xyrrolin, and cyclopiazonic acid

**Scheme 3:**
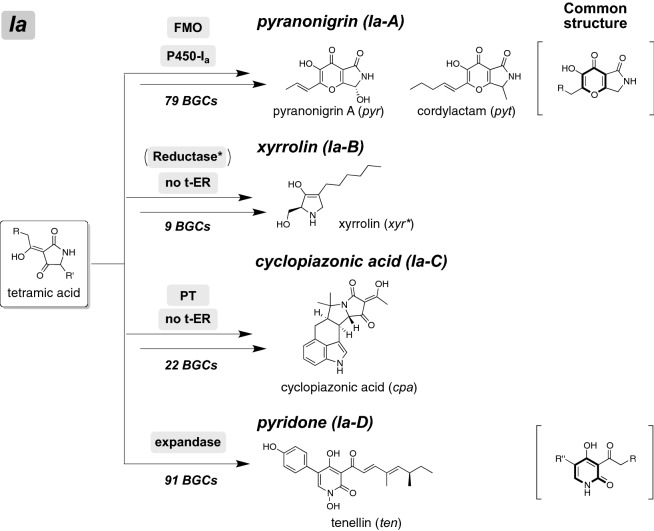
Proposed biosynthetic pathways of hybrid NPs produced by four NP clades; pyranonigrin, xyllorin, cyclopiazonic acid, and pyridone. The key modification enzymes involved in synthesizing each core are denoted in boldface. Detailed biosynthetic schemes are summarized in Supplementary Information.

The successful identification of three NP clades, tenellin, leporin, and illicicolin, suggested that the modification enzyme (ME) search especially focusing on key modification enzymes, which synthesize a characteristic structure of hybrid NP, might be effective to identify other NP clades. According to this hypothesis, we examined the ME search focusing on other BGCs found in Fig. 2 and identified three NP clades, pyranonigrin (Ia-A), xyrrolin (Ia-B), and cyclopiazonic acid (Ia-C). For pyranonigrin clade identification, we selected two key modification enzymes (MEs), FMO and P450, by considering the biosynthetic pathway of pyranonigrins (Schemes 3 and S2, Fig. 1, and Table S2)^26–28^. A local BLAST search of FMO/P450 using our hybrid library (884 BGCs) identified 79 BGCs (identities > 40%), which are most likely responsible for the production of various pyranonigrins. Interestingly, 70% of the strains with these pyranonigrin BGCs belong to only four fungal genera. Similarly, we identified the xyrrolin^29^ and cyclopiazonic acid^30^ clades, consisting of 9 and 22 BGCs respectively, which might synthesize xyrrolin- and cyclopiazonic acid-related hybrid NPs (Schemes 3 and S2, Fig. 1, and Table S2).

The borders of identified NP clades are almost identical to the apparent borders of the phylogenetic tree (Fig. 2). Based on this finding, we re-examined 121 unclassified hybrids and identified several distinct clades composed of less than 25 hybrids. Assuming that they were unclassified NP clades, we checked the MEs in the corresponding BGCs by using a local BLAST search and the gene cluster search programs, such as SMURF and 2nd Find, to identify conserved modification enzyme genes (Table S6). As a result, we proposed five additional NP clades (Fig. 2 and Table S2): Ia-α (numbers of BGCs; 16), Ia-β (17), Ia-γ (14), Ia-δ (10), and Ia-ε (20). The location of each predicted clade is described in Fig. 2. In most cases, we could not predict the hybrid NPs produced by the BGCs in these new clades. An exceptional case was the BGCs in clade Ia-δ, which possessed only a trans-ER gene as the key ME. Given the fact that trans-ER is essential in synthesizing a polyketide chain with a skipped polyene structure, we hypothesized that the BGCs in clade Ia-δ produce tetramic acid derivatives with a skipped polyene side chain such as penicillenols (Scheme S1).

#### Key modification enzymes used for the ME search

**Figure 3:**
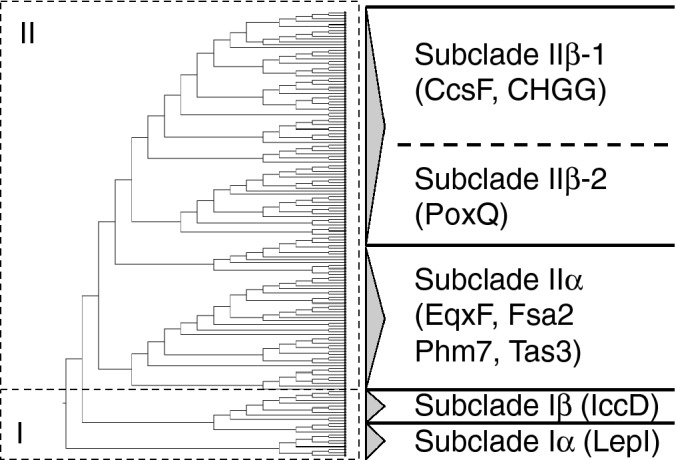
Phylogenetic tree of DAases. Two major DA clades, I and II, are further divided into subclades, Iα, Iβ, IIα, IIβ-1, and IIβ-2, which correlate with the function of the characterized DAases. The accession numbers of the DAases used in this phylogenetic analysis are summarized in Table S5.

The results described above showed that identification of NP clades by the ME search requires restricted number of modification enzyme, which are summarized in Table 1. Among them, Diels-Alderase (DAase), which was found in almost 20% of the BGCs in our dataset, is one of the key enzymes involved in the skeletal construction during hybrid NP biosynthesis^31^. Pfam analysis of functionally characterized DAases revealed that they could be divided into two groups: DAases with an *S*-adenosyl-l-methionine (SAM) binding motif and those with no characteristic motif. The former type-I DAases are only found in PKS–NRPS clades, leporin and illicicolin, and mediate either the hetero-Diels–Alder reaction or the inverse electron demand Diels–Alder reaction^23,25^. Other type-II DAases^31–37^ that catalyse the [4 + 2] cycloaddition to afford either a decalin or a macrocyclic ring are distributed in PKS–NRPS clades Ib and III. Our phylogenetic analysis of putative DAases revealed that DAases comprise two different clades: I (for type-1 DAases) and II (for type-2 DAases) (Figs. 3 and S2, Table S6). Additionally, we found that both type-I and type-II DAases can be further divided into two subclades: clade Iα functionally characterized DAase: LepI^25^), Iβ (IccD^23^), IIα (EqxF^31^, Fsa2^32^, Phm7^32^, and Tas3^33^), and IIβ (CcsF^34^, CHGG_01241^35^, PoxQ^36^, and MycB^37^). IIβ can be further divided into two groups, which reflect the two alternative functions of synthesizing either a macrocyclic (IIβ-1) or decalin (IIβ-2) ring. This classification of DAases enabled us to use them for the ME search.

**Table 1:** Key modification enzymes involved in the functionalization of intermediates produced by hybrids.

NP subclade	Ia-A	Ia-B	Ia-C	Ia-D-1	Ia-D-2	Ia-D-3	Ib-A	Ib-B	Ib-C
Knoevenagelase (Level I)	−	−	−	−	−	−	−	−	−
t-ER (Level I)	−	−	−	+	+	+/−	+	+/−	+
Key ME (Level II)	FMO	Reductase	PT	Expandase (P450)	Expandase (P450)	Expandase (P450)	P450 (Ib)	Aldolase	DAase (type IIα)
	P450 (Ia)	−	−	−	DAase (type Iα)	DAase (type Iβ)	−	trans-aminase	−
	−	−	−	−	Reductase	−	−	−	−
Representative hybrid NP	Pyranonigrin	Xyrroline	Cyclopiazonic acid	Tenellin	Leporin	Illicicolin	Burnettramic acid A	Sch210972	Equisetin

NP subclade	Ib-D	III-B	II-A	II-B	II-C	II-D	III-A	III-C	III-D
Knoevenagelase (Level I)	−	−	+	+	+	+	+	+	+
t-ER (Level I)	−	+	−	+	+	−	+	+	+
Key ME (Level II)	−	P450 (III)	MT	P450 (IIB)	Mono-oxygenase	P450 (IID)	DAase (type IIβ)	DAase (type IIβ)	DAase (type IIβ)
	−	α-KG	P450 (IIA)	−	−	−	−	Trans-aminase	−
Representative hybrid NP	Fusaridione	Acyltetronic acid	Fusarin	Himeic acid	Flavipucine	Pseurotin	Chaetoglobosin	Oxaleimide	Cytochalasin

#### Identification of other NP clades for tetramic acid derivatives

**Scheme 4:**
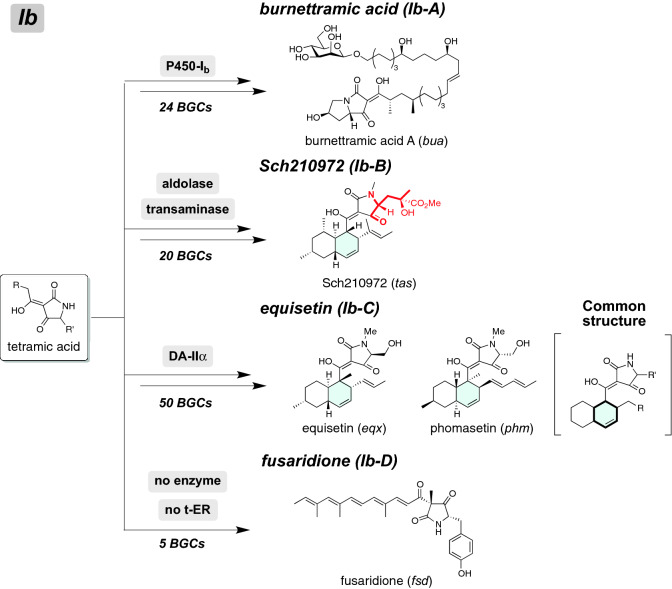
Proposed biosynthetic pathways of hybrid NPs produced by BGCs of four NP clades; burnettramic acid, Sch210972, equisetin, and fusaridione. Detailed biosynthetic schemes are summarized in Supplementary Information.

Six identified NP clades described above include BGCs for synthesizing hybrid NPs derived from a tetramic acid intermediate. The tetramic acid skeleton is also found in other hybrid NPs such as equisetin, of which hybrids form other clades in the phylogeny (Clade Ib). The ME search focusing on these hybrids resulted in identification of seven hypothetical NP clades: burnettramic acid (characterized hybrid: BuaA^38,39^, number of hybrids: 24), Sch210972 (TasS^40^, 20), equisetin (EqxS^41^, 50), fusaridione (FsdS^41^, 5), Ib-α (25), Ib-β (12), and Ib-γ (16) (Schemes 4 and S3, Tables S3 and S6). Hybrid NPs classified in this clade have unique polyketide side chains or moieties on the common tetramic acid moiety. Burnettramic acid A (final classification: Ib-A) has a saturated polyketide chain possessing three hydroxyl groups, whereas fusaridione (Ib-D) has a polyene side chain. Among them, burnettramic acid A is a new class of hybrid NPs, which consists of β-D-mannose linked to a pyrrolizidionedione unit via a 26-carbon chain. Our phylogeny-based analysis revealed 24 homologous hybrids, most of which accompany a cytochrome P450 gene. Considering that structurally related epicoccamides have only one hydroxyl group at the terminal position of the polyketide chain (Scheme S3), P450 might be responsible for the hydroxylation. Extended ME analysis showed that seven BGCs including that for burnettramic acid A possess a putative glycosyltransferase, which most likely catalyzes a glycosyltransfer reaction (Scheme S3)^38^. In contrast, equisetin (Ib-C) has a decalin skeleton, which undergoes no additional functionalizations during biosynthesis. Of particular note are the hybrid NPs, such as Sch210972 (Ib-B), which contain a nonproteinogenic amino acid, 4-hydroxyl-4-methyl glutamate (4-HMG). Synthesis of 4-HMG is catalysed by an aldolase and transaminase from two units of 4-hydroxy-4-methyl-2-oxoglutarate (Scheme S3)^40^, thereby allowing us to use them for the ME search. 4-HMG is also found in known hybrid NPs such as harzianic acid, which possesses a linear polyketide chain instead of the decalin skeleton. Interestingly, the Sch210972 BGC group possesses a distinct DAase gene, although these are minor in the identified BGCs. BGCs lacking the DAase gene were found in the Sch210972 clade, suggesting that they might produce harzianic acid related hybrid NPs.

#### Identification of NP clades for pyrrolinone derivatives

**Scheme 5:**
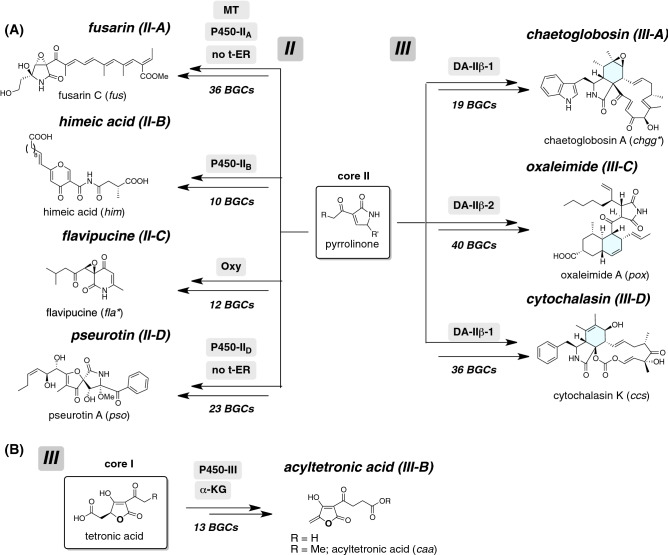
Proposed biosynthetic pathways of hybrid NPs produced by BGCs of NP clades of (**A**) pyrrolinone containing hybrid NPs and (**B**) tetronic acid derivatives. Asterisk indicates tentative BGC names for chaetoglobosin (*chgg*) and flavipucine (*fla*). Detailed biosynthetic schemes are summarized in Supplementary Information.

Different location of clades Ia and Ib in the hybrid phylogeny highlights the presence of new clades II and III (Fig. 1). Most functionally characterized hybrids found in these clades are involved in the biosynthesis of hybrid NPs derived from a pyrrolinone intermediate. The ME search for these BGCs allowed us to identify eight NP clades (Schemes 5, and S4–S6, Tables S4 and S5): the fusarin^42^ clade (II-A, number of BGCs: 36), himeic acid^43^ clade (II-B, 10), flavipucine^44^ clade (II-C, 12), pseurotin^45^ clade (II-D, 23), chaetoglobosin^35^ clade (III-A, 19), acyltetronic acid^46^ clade (III-B, 13), oxaleimide^36^ clade (III-C, 40), and cytochalasin^47^ clade (III-D, 36). Hybrid NPs except acyltetronic acid are commonly derived from a pyrrolinone intermediate possessing a linear polyketide chain. The pyrrolinone skeleton is conserved in fusarin and pseurotin, which undergo modification reactions on the polyketide chain. In contrast, the pyrrolinone skeleton of himeic acid and flavipucine is subjected to oxidative rearrangement to afford a six-membered heterocyclic ring. Three hybrid NPs such as chaetoglobosin, oxaleimide, and cytochalasin, are unique in that they have polycyclic structures. As an exception, acyltetronic acid has a tetronic acid moiety instead of the tetramic acid/pyrrolinone found in other hybrid NPs.

We could also predict three NP clades flanked by the above NP clades via the ME search (Table S7): II-α (numbers of the hybrid; 11), II-β (18), and II-γ (18). In the case of clade III, the identified NP clades occupy only 43%. Most functionally unknown hybrids are phylogenetically related to CaaA (Table S5), which produces a tetronic acid core instead of a tetramic acid core. A lack of sufficient understanding regarding their biosynthesis prevented further detailed predictions.

Overall, the analysis revealed 29 NP clades (74%, 660 BGCs); 18 NP clades include BGCs of known hybrid NPs, and the remaining 11 NP clades are composed of functionally unknown BGCs (Table 2). Given the fact that BGCs that produce structurally related hybrid NPs are classified into the same NP clade, we could predict 29 NP groups through the phylogenetic analysis of the hybrid genes as well as the ME search of the corresponding BGC. An exceptional example is UcsA^48^, which participates in the biosynthesis of UCS1025A. We initially expected that UcsA would be classified into clade III-C based on the biosynthetic relationship between UCS1025A and oxaleimide (Schemes S5 and S7). However, our analysis revealed that UcsA forms a distinct small clade comprising only three hybrids. This unexpected result suggests that close attention is needed when discussing the chemical space of hybrid NPs.

**Table 2:** Summary of bioinformatics analysis of 884 BGCs.

Clade	Known NP groups (%, number of BGCs)	Predicted NP groups (%, number of BGCs)	Not classified (%, number of BGCs)
Ia	6 (64%, 201)	5 (25%, 77)	(11%, 35)
Ib	4 (52%, 99)	3 (25%, 47)	(23%, 43)
II	4 (63%, 81)	3 (37%, 47)	(0%, 0)
III	4 (43%, 108)	Not examined	(57%, 146)
Total	18 (53%, 489)	11 (21%, 171)	(26%, 224)

#### Two alternative pathways leading to common biosynthetic intermediates

**Scheme 6:**
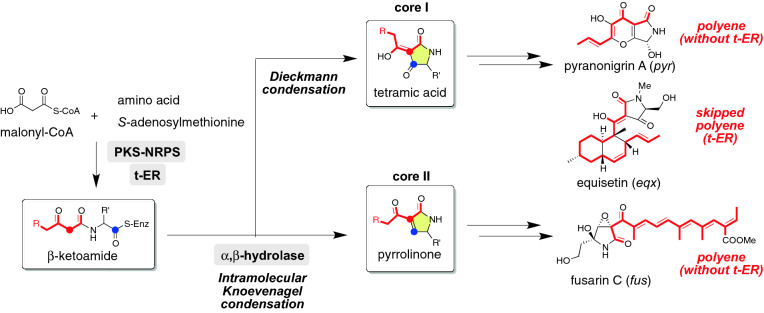
Two alternative modifications of the β-ketoamide intermediate leading to two different cores, I and II.

The known hybrid NPs described above are biosynthesized via either a tetramic acid (core-I) or a pyrrolinone (core-II), which commonly possess a five-membered heterocyclic ring (Scheme 6). In the former case, a single DKC domain directly catalyses a Dieckmann condensation to give a tetramic acid intermediate^49^. In the latter case, the R domain most likely mediates a reductive cleavage to give an aldehyde intermediate, which is then subjected to a Knoevenagel condensation through the action of an α,β-hydrolase. From the biosynthetic point of view, function of the terminal off-loading domains is crucial for distinguishing biosynthetic pathways leading to core-I and -II. Based on the proposal that key polar amino acid residues are conserved in the DKC domain^50^, we employed exhaustive bioinformatics analysis of these domains. However, we failed to identify such residues specifically in the DKC domain. Therefore, in this paper, we focused on the α,β-hydrolase gene, which is called as “Knoevenagelase”. The involvement of putative Knoevenagelase (pKN) in pyrrolinone formation is supported by a gene deletion experiment for the homologous enzyme gene (*fus2*) found in fusarin BGC^42^, and by heterologous expression experiments of hybrid and trans-ER genes (PKS-NRPS/trans-ER; *ccsA*/*C* and *aceI*/*RAP1*)^51,52^, though in vitro analysis of pKN have not been reported. Our comprehensive ME search described above revealed that the pKN gene is highly conserved in the BGCs of 7 NP clades, which may be involved in the biosynthesis of hybrid NPs possessing the pyrrolinone skeleton (Scheme 5A). On the other hand, BGCs of the remaining 9 NP clades for tetramic acid containing hybrid NPs lack the pKN gene (Schemes 3, 4, and 5B). These circumstantial evidences support the importance of the pKN in the construction of the pyrrolinone skeleton.

### Hierarchical classification of fungal hybrid BGCs

The phylogenetic and BGC analysis described above shows that the hybrid phylogeny includes sufficient biosynthetic information for predicting the chemical structure of the hybrid NPs. To understand the phylogeny of the hybrids, we propose hierarchical classifications focusing on the pKN and key ME, reflecting the early and late stage biosynthesis of hybrid NPs. The Level I classification focusing on the pKN gene revealed four major clades, Ia, Ib, II, and III (Fig. 1). Clades Ia and Ib are composed of NP clades, in which BGCs lack the Knoevenagelase gene. On the other hand, clade II is formed by NP clades, which include BGCs possessing the Knoevenagelase gene (Fig. 1). Clade III is unique in that relatively small BGC groups possessing or lacking the Knoevenagelase gene were mixed. Level II classification using the key modification enzymes resulted in identification of 29 NP clades. Notably, hybrid NPs classified into the same NP clade have a common chemical structure. Therefore, we can easily predict structural novelty by checking the NP clades of a target hybrid. This prediction is difficult by using up-to-date methods for the simple analysis of the entire fungal BGC.

## Discussion

Predicting the chemical structure of NPs using bioinformatics analysis based on genomic data is one of the biggest challenges in natural product research. As they are different from bacterial multi-modular PKS, the domain organization of which reflects its structure, fungal HR-PKSs have iteratively acting single modules, thereby preventing speculation of the NP chemical structure. To address this problem, we proposed the knowledge-based classification of fungal NPs, because experimental data have been accumulated on the functional analysis of HR-PKS and hybrids. After the phylogenetic analysis of the hybrids, we performed an extensive comparative analysis of the target BGCs with key modification enzymes whose functions have been experimentally characterized. These analytical data enabled us to classify the hybrids into 29 groups, including 11 experimentally uncharacterized groups (Table 2). The number of predicted NP groups was relatively smaller than expected. Assuming that a single NP group produces more than 100 derivatives on average^13–16,53^, based on a previous report that cytochalasans, one of the largest families of hybrid NPs, comprise over 300 derivatives^15^, we speculated that 660 BGCs classified into 29 groups could potentially generate about 2,900 NPs. We are not sure regarding how many unique fungal strains exist in nature, but could estimate an approximate number of fungal hybrid NPs based on our analysis (Fig. 1). In addition to the 29 NP clades, small clades are found in the phylogeny, represented by a putative clade composed of the hybrid for UCS1025A. At present, we can speculate at least two reasons for this; (1) this clade is in the middle of growing or (2) the genome sequencing of fungi is biased toward phytopathogenic fungi and thus does not cover fungi uniformly. Further genome analysis of taxonomically unique fungi is essential to answer this question.

Representative hybrid NP cytochalasans including chaetoglobosins and cytochalasins are widely distributed in various fungi belonging to more than 20 genera and 4 subphyla^14^. This is consistent with our results finding numerous cytochalasan BGCs in our analysis (Table S5). These closely related hybrid BGCs commonly possess highly homologous genes of both hybrid (*ccsA*) and modification enzyme genes (*ccsC/E/F*), suggesting that these homologous genes are co-evolved to produce closely related NPs such as aspochalasin, phomacin, and alachalasin (Scheme S5). The diverse distribution of structurally related cytochalasans in fungi indicated that when considering their biological activities, molecular evolution might occur through natural selection, which may be important for their survival or life cycle. Indeed*,* cytochalasin is known as a virulence factor that blocks cytokinesis in some producers^54^. These potent biological activities make producers more prosperous in increasing their population. This property might thus contribute to increasing the probability of horizontal gene transfer (HGT) through Ascomycetes, which may be one of the reasons explaining the diverse distribution of cytochalasans in fungi.

Fungal iterative PKS can generate a large number of diverse molecular skeletons via its exquisite control of the catalytic process. Additional modifications can diversify the final products. Representative PK backbones of known hybrid NPs are summarized in Figure S1. Most PK backbones are composed of less than the C18 carbon chain as in the case of a fatty acid (C18: stearic acid). Their structural diversity is restricted in the numbers and positions of the methyl and olefin functionalities. This suggests that hybrid PKS limits the PK structure itself, although they can theoretically create diverse structures.

Among several late-stage modifications discussed in the above sections, intramolecular [4 + 2] cycloadditions constructing a polycyclic skeleton frequently takes place in the biosynthesis of fungal hybrid NPs. One of the major reasons is that the skipped polyene possessing diene- and dienophile-moieties can be inherently installed through the action of the hybrid and trans-ER (Scheme S1). This is consistent with our results that almost 20% of the hybrid genes accompany the DAase genes (Tables S2, S3, S4, S5). Despite the fact that they are key enzymes in hybrid NP biosynthesis, to our knowledge, no detailed bioinformatics analysis has been reported. Our phylogenetic analysis of the DAases revealed that they are divided into four clades (Fig. 3). Of particular importance, this classification reflects the function of DAases, thus allowing us to use them for Level II classification. As in the case of cytochalasans, the frequent occurrence of many [4 + 2]-adducts indicates that their rigid molecular skeleton might contribute to their remarkable biological activities.

In this study, we propose a promising bioinformatics approach to predict the chemical structure derived from a target BGC possessing the hybrid gene. This prediction can be applied to the dereplication method for the differentiation of novel or known hybrid NPs, the genome mining of novel NPs by expressing candidate BGCs, and the construction of a focused library of structurally related hybrid NPs. The wide distribution of a specific class of hybrid NPs in taxonomically different fungi suggests the involvement of HGT among Ascomycetes. We anticipate that this bioinformatics approach can be applied to other classes of NPs, providing opportunities to examine their chemical space.

## Methods

### Data collection

Publically available fungi assembly data used in this study are obtained from the National Center for Biootechnology Information (NCBI) using an File Transfer Protocol (FTP) site. Protein sequence analysis was completed in 1,462 assemblies within the collected date (4,922 assemblies). Those including 14,493,696 proteins were used for identification of the hybrids.

### Hybrid identification

To retrieve the hybrids from each assembly, Pfam motif search focusing on KS [PF00109], AT [PF00698], and C [PF00668] domains was conducted. The resulting dataset_1 includes both the hybrid gene and LovC type HR-PKS gene. Proteins possessing more than 3,000 a.a. were then extracted to construct dataset_2 excluding LovC type HR-PKS. Subsequently, highly homologous hybrids within taxonomically related fungi were excluded to afford dataset_3. The criteria of the evolutionary distance used to predict proteins as orthologs were set less than 0.05. Finally, functionally characterized hybrids were manually selected and added to the dataset_3 to construct final dataset composing of 884 hybrids. All accession numbers of the hybrids and the related information including strain name were provided in Supplementary Tables S2, S3, S4 and S5.

### Genealogy construction

The hybrids, NR-PKSs from fungi, and FASs were aligned using the MAFFT online service (version 7) for multiple sequence alignment (FFT-NS-2 algorithm). Construction of phylogenetic tree was based on the average linkage (UPGMA) method. Visualization was conducted with FigTree (version 1.4.3).

### BGC analysis

The amino acid sequences of key modification enzymes were manually collected from public database. The homologous proteins were extracted by Local BLAST search using BlastStation (TM Software, Inc.). The results were summarized in Supplementary Tables S2, S3, S4 and S5.

## Supplementary information

Supplementary Information.

Supplementary Table S2.

Supplementary Table S3.

Supplementary Table S4.

Supplementary Table S5.

Supplementary Table S6.
