# Supplementary material for: Predicting the chemical space of fungal polyketides by phylogeny-based bioinformatics analysis of polyketide synthase-nonribosomal peptide synthetase and its modification enzymes
Source: Sci Rep. 2020 Aug 11;10:13556. doi: 10.1038/s41598-020-70177-w (PMC7421883; doi:10.1038/s41598-020-70177-w)
Supplement: Supplementary file 1 — Supplementary Information. [file 41598_2020_70177_MOESM1_ESM.docx]

**Supporting Information**

**Predicting the chemical space of fungal polyketides by phylogeny-based bioinformatics analysis of polyketide synthase-nonribosomal peptide synthetase and its modification enzymes**

Atsushi Minami*, Takahiro Ugai, Taro Ozaki, and Hideaki Oikawa*.

**Table of Contents**

**Figures**

Figure S1 : PK backbone structures of hybrid NPs. Page S2

Figure S2 : Multiple amino acid sequence alignment of selected DAases. Page S4

**Schemes**

Scheme S1 : Proposed biosynthetic pathway of 2-pyridone containing hybrid NPs. Page S5

Scheme S2 : Proposed biosynthetic pathway of clade Ia_hybrid NPs. Page S7

Scheme S3 : Proposed biosynthetic pathway of clade Ib_hybrid NPs. Page S8

Scheme S4 : Proposed biosynthetic pathway of clade II_hybrid NPs. Page S10

Scheme S5 : Proposed biosynthetic pathway of clade III_hybrid NPs. Page S11

Scheme S6 : Proposed biosynthetic pathway of acyltetronic acid. Page S12

Scheme S7 : Proposed biosynthetic pathway of Ucs1025A. Page S12

**Tables**

Table S1 : FAS and NR-PKS used for the phylogenetic analysis Page S13

Table S2-S6 : See attached excel files.

Table S7 : Key modification enzymes conserved in hypothetical NP clades. Page S13

**Figure S1.** PK backbone structures of hybrid NPs.

**Figure S1.**

**Figure S2.** Multiple amino acid sequence alignment of selected DAases. Highly conserved amino acid residues in type IIα and IIβ DAases are described in red bold characters.

OAK93956 INSTNW**E**Q**W**EF**D**GLSHTGLSSVLLVFSRDPSYAFFGQGNLRVEFYIVFGDG--TRIEALD 137 **PoxQ** LNATAG**E**Q**W**AF**D**GTSSSGRSGLLLGIYRDASYAFLGPGNFRLSLDLVWDNS--TTWSTVD 153 PWY66094 LNATAG**E**Q**W**AF**D**GTSSSGRSGLLLGIYRDASYAFLGPGNLRLSLDLVWDNG--TTWSTVD 99 TLD18460 LNSTGG**E**Q**W**AF**D**GVSEDGLNSFIFGFYRDPNYSILGAGNFRLSLEFGFAER--ERFAELY 88 TLD08746 LNSTGG**E**Q**W**AF**D**GVAEDGVQSFMFGFYRDPNYSILGAGNFRLSLEFGFANR--ERVAELY 112 Ace1_DA LNSTGG**E**Q**W**AF**D**GVSEDGVKSFMFGFYRDPNYSILGAGNFRLSLEFGFANR--ERVAELY 112 TLD07679 LNSSGG**E**Q**W**EF**D**GVSEDGMQSFIFGFYRDPNYAILGTGNLRVSIEFGFEDR--TRFSEVY 148 **CcsF** LNSTAG**E**Q**W**EF**D**GVSEDGMQSFVFGFYRDPNYAILGTGNFRLSIEFAFANR--TRFYEVY 136 RDL33254 LNSSAG**E**Q**W**EF**D**GVSEDGMQSFIFGFYRDPNYAILGAGNFRLSVEFAFADR--TRFYEVY 147 RDL40497 LNSSSG**E**Q**W**EF**D**GVSDDGMFSFIFGFYRDPSLSLMGSGNFRLSAELAYANG--TRFGRVD 141 EHK20207 LNSTGG**E**Q**W**EF**D**GVSDDGLMAFCFGFYRDPNYAILGSGNLRLSAEFSRVNQ--ERFMRVD 136 **CHGG_DA** LNSTGG**E**Q**W**EF**D**GVSEDGQMAFCFGFYRDPNYAILGTGNLRLSAEFSRPNK--TRFVRVD 140 GAW16229 MNSTAG**E**Q**W**EF**D**GVSEDGTRAFIFGVYRDPNYAFLGTGNLRAYVEFVFDDG--SRYAVVD 151 EAQ88667_DA MNSTAG**E**Q**W**EF**D**GVSEDGMQAFVFGFYRDPNFSFFGAGNLRVYAEFAFANG--SRYAIVD 133 EOO03341 MNETAG**E**Q**W**EF**D**AVSDDGLSGVILGFYRDPNYSVLGSGNLRMYIEIAFPEPGRPRFVQID 85 **Tas3** IRKTAV**E**V**W**LF**D**AIAEDGSSAITISFFRDALA---APAGFRIAVNASWSDG--TIWGKPL 94 OTA57457 FNKAAV**E**V**W**LF**D**AMTTDGKTAFTVSFVRDVLA---APAGFRIQVNATWPDG--TKWSNPL 94 **Phm7** ISVDSW**E**L**W**EF**D**TFDTNGSVAFGCSLYRDARG--VEQGGFHAEVNALWPDG--THWGETL 97 RZR69978 IKETGW**E**L**W**FV**D**GVSAEEKAAITIGLSRNGED--RTHNGFKAQITAIWPDE--STWYRDL 96 **Fsa2** IPKTAW**E**L**W**YF**D**GISKDDKSSIVIGVTRNAEG--LKHGGFKVQVFVIWADE--RTWHRDL 95 **EqxF** IPKTAW**E**L**W**YF**D**GISKDDRSSIVIGVTRNAEG--LKHGGFKVQVFVIWADE--RTWHRDL 95

OAK93956.1 TDLF---DGE--VKGKYRDKNTAHHLQFVSPA-NDKS**W**E**F**QVDHMLTH**Y**EFSAGG----G 371 **PoxQ** RPVR---M-EKQA------RETGYEVDLVSPA-QGRR**W**V**F**GLEYRNQE**F**EFELGD----A 378 PWY66094 RPVR---G-EGQERERETETETGYEVELVSPG-QGRR**W**V**F**GLEYRNQE**F**EFELGE----T 342 TLD18460 EKTY---GGT--VTGTLKDKVTGFQIELVSPS-NKRH**Y**T**F**FVEHKNLA**F**EYLLGE----G 320 TLD08746 QKTY---GGA--VTGDLKDKVTGFQVELVSPS-KKRH**Y**T**F**FIEHKNLA**F**EYLLGE----G 344 Ace1_DA QKTY---GGA--VTGDLKDKVTGFQVELVSPS-KKQH**Y**T**F**FIEHKNLA**F**EYLLGE----G 344 TLD07679 TKTY---GGA--TTGTLKDKVTGFQLELVSPS-KMRH**Y**T**F**FVEHLNVG**F**EYILGE----G 380 **CcsF** SKTY---GGA--VTGTLKDKVTGYQLELVSPG-RMQH**Y**T**F**FVEHANLG**F**EYILGE----G 368 RDL33254 TKTY---GGS--VTGTLKDKVTGFQLELVSSA-TMQH**Y**T**F**FVDHANLG**F**EYILGE----G 380 RDL40497 TKTY---GGT--VYGTLRDKVTGYELELISPR-KKKH**W**T**F**IMEHKNIA**F**EYLIGN----G 371 EHK20207 EKTY---GGK--VTGSLKDKVTGFELVMVSPK-SKDQ**W**S**F**IITNEGVG**F**EYMLGE----G 366 **CHGG_DA** TKTY---GGR--VSGNLKEKATGYELVMVSPS-AKKQ**W**S**F**SITNEAIG**F**EYMLGE----G 370 GAW16229 RKLY---GGEGITTEHLEDRVTGIEICLEQPN-QQLA**W**V**F**SVVFKKVG**F**EYVLTE----G 395 EAQ88667_DA RKLY---AGQGITTEALGDKVTGMELVLVSPS-RNRR**W**G**F**VLTHKNLA**F**EYNLGS----G 366 EOO03341 QKVY---GGEGISG-SLSDKATGFVLDLVSPS-QGKQ**W**T**F**LITHRNLV**F**EYFLGG----G 322 **Tas3** KKLF---EGEG-LPAPFRHQNVGYQLEYTSGGPDGKT**W**V**F**ETRHQRAW**Y**RKPTGP----G 363 OTA57457.1 GKLY---EGEG-VPAVFRHKNVGCRIEFRSGGPNGQR**W**S**F**DARHHRAW**W**SKPSSPPGPNA 353 **Phm7** TKVQPDEKSQG-LSGKFRDGNVGYVLEFAKKD-SEHG**W**T**F**QISHKRAV**W**SEPTSAPGPDG 353 RZR69978.1 RKVYDDANHSG-RTGAFRDHNKGYIVRFIKAG-TGEH**W**E**F**QVHHDRIF**W**NIPTSAPGPNA 347 **Fsa2** SKQDNSDSEDV-VTGGYRDKNTGYTVEFVEKGNEGQR**W**K**F**QVRHERII**W**NTPTSRPGPDA 343 **EqxF** SKQNTSDSGDA-VTGEYRDKNTGYTVEFVGKGNEEQR**W**E**F**QVRHERII**W**NTPTSRPGPDA 346

**Scheme S1.** Proposed biosynthetic pathway of (A) tenellin (Ia-D-1), (B) leporin (Ia-D-2), (C) illicicolin (Ia-D-3). Known hybrid NPs possibly produced by each clade are enclosed by dotted line [S1]. (D) Other known hybrid NPs possibly produced by BGCs classified in pyridone clade (Ia-D).

**Scheme S1.** Continued.

**Scheme S2.** Proposed biosynthetic pathway of (A) pyranonigrins (Ia-A), (B) xyrrolin (Ia-B), and (C) cyclopiazonic acid (Ia-C). Known hybrid NPs possibly produced by each clade are enclosed by dotted line [S2-S5].

**Scheme S3.** Proposed biosynthetic pathway of (A) burnettramic acid A (Ib-A), (B) equisetin (Ib-B-2), (C) Sch210972 (Ib-B-1), and (D) fusaridione (Ib-C). Known hybrid NPs possibly produced by each clade are enclosed by dotted line [S6-S7].

**Scheme S3.**

**Scheme S4.** Proposed biosynthetic pathway of (A) fusarin (II-A), (B) himeic acid (II-B), (C) flavipucine (II-C), and (D) pseurotins (II-D). Known hybrid NPs possibly produced by each clade are enclosed by dotted line [S8, S9].

**Scheme S5.** Proposed biosynthetic pathway of (A) chaetoglobosin (III-A), (B) cytochalasin (III-C), (C) oxaleimide (III-C), and (D) Ucs1025A. Known hybrid NPs possibly produced by each clade are enclosed by dotted line [S10-S12].

**Scheme S5.** Continued.

**Scheme S6.** Proposed biosynthetic pathway of acyltetronic acid.

**Scheme S7.** Proposed biosynthetic pathway of Ucs1025A.

**Table S1.** FAS and NR-PKS genes used for the phylogenetic analysis.

**Table S2 to S6.** See attached excel files.

**Table S7.** Key modification enzymes conserved in hypothetical NP clades.

**References**

S1. Jessen, H. J. & Gedemann K. 4-Hydroxy-2-pyridone alkaloids: Structures and synthetic approaches. *Nat. Prod. Rep.*, **27**, 1168-1185 (2010).

S2. Isaka, M., Chinthanom, P., Rachtawee, P., Somyong. W., Luangsaard, J. J. & Hywel-Jones, N. L. Cordylactam, a new alkaloid from the spider pathogenic fungus Cordyceps sp. BCC 12671. *Phytochem. Lett.*, **6**, 162-164 (2013).

S3. Schlingmann, G., Taniguchi, T., He, H., Bigelis, R., Yang, H. Y., Koehn, F. E., Carter, G. T. & Berova, N. Reassessing the structure of pyranonigrin. *J. Nat. Prod.*, **70**, 1180-1187 (2007).

S4. Yokoyama, M., Hirayama, Y., Yamamoto, T., Kishimoto, S., Tsunematsu, Y. & Watanabe, K. Integration of chemical, genetic, and bioinformatics approaches delineates fungal polyketide-peptide hybrid biosynthesis. *Org. Lett.*, **19**, 2002-2005 (2017).

S5. Sandmeier P & Tamm C. New spirostaphylotrichins from Staphylotrichum coccosporum. *Helv Chim Acta*, **72**, 784–792 (1989).

S6. Jiang, M. –Y., Feng, T. & Liu, J. –K. N-containing compounds of macromycetes. *Nat. Prod. Rep.*, **28**, 783-808 (2011).

S7. Mo, X., Li. Q. & Ju, J. Naturally occurring tetramic acid products: isolation, structure elucidation and biological activity. *RSC Adv.*, **4**, 50566-50593 (2014).

S8. Sugawara, T., Shinonaga, H., Yoji, S., Yoshikawa, R. & Yamamoto, K. Polyene-based compounds. *Jpn Patent* 319289, 1996.

S9. Jo, D. & Han, S. Total syntheses of spirocyclic PKS-NRPS-based fungal metabolites. *Chem. Commun.*, **54**, 6750-6758 (2018).

S10. Scherlach, K., Boettger, D., Remme, N. & Hertweck, C. The chemistry and biology of cytochalasans. *Nat. Prod. Rep.*, **27**, 869-886 (2010).

S11. Skellam, E. The biosynthesis of cytochalasans. *Nat. Prod. Rep.*, **34**, 1252-1263 (2017).

S12. Li, G., Kusari, S. & Spiteller, M. Natural products containing ‘decalin’ motif in microorganisms. *Nat, Prod. Rep.*, **31**, 1175-1201 (2014).
